# Ginkgolic acids inhibit migration in breast cancer cells by inhibition of NEMO sumoylation and NF-κB activity

**DOI:** 10.18632/oncotarget.16626

**Published:** 2017-03-28

**Authors:** Sami Hamdoun, Thomas Efferth

**Affiliations:** ^1^ Department of Pharmaceutical Biology, Institute of Pharmacy and Biochemistry, Johannes Gutenberg University, Mainz, Germany

**Keywords:** breast cancer, ginkgo biloba, NEMO, NF-κB, sumoylation

## Abstract

Ginkgolic acids (GA), a group of alkyl phenols found in crude extracts of *Ginkgo biloba* leaves, are known to have anticancer activity, but their mode of action is not well understood. Our aim in this study was to investigate the anti-migratory activity of seven GA against breast cancer cells and to determine the molecular mechanism behind this activity. All seven GA and their mixture inhibited wound healing in MCF-7 and MDA-MB 231 breast cancer cells. None of the compounds nor the mixture showed cytotoxicity towards the two cell lines, if tested by the resazurin assay. C13:0 inhibited NF-κB activity in the HEK Blue Null 1 reporter cell line. Furthermore, C13:0 inhibited degradation of nuclear factor of κ-light polypeptide gene enhancer in B-cells inhibitor α (IκBα). Sumoylation assay revealed that GA inhibited sumoylation of NF-κB essential modulator (NEMO). Molecular docking on SUMO-activating enzyme E1 showed that the seven GA bound to the active adenylation site with high calculated affinities ranging from -10.28 to -12.27 kcal/mol. Quantitative RT-PCR using C15:0, C13:0 and the mixture showed a significant down-regulation of urokinase plasminogen activator (uPA), plasminogen activator inhibitor-1 (PAI-1), C-X-C chemokine receptor type 4 (CXCR4) and matrix metalloproteinase 9 (MMP-9). We conclude that GA revealed considerable anti-migratory activity at non-cytotoxic concentrations, indicating anti-metastatic activity with low toxicity. This effect can be explained by the inhibition of NEMO sumoylation leading to inhibition of IκBα degradation and consequently a reduction of NF-κB activity, leading to the down-regulation of metastasis related genes including uPA, PAI-1, CXCR4, and MMP-9.

## INTRODUCTION

*Ginkgo biloba* L. has been found in petrified fossils dating back more than 200 million years, and it is therefore referred to as a living fossil [1]. The use of *Ginkgo biloba* in the treatment of diseases has been documented 2800 years ago in traditional Chinese medicine. Until now, *Ginkgo biloba* is still used in Chinese medicine to treat cardiovascular diseases as well as pulmonary diseases, including bronchitis and asthma [2]. The use of extract from *Ginkgo biloba* for treatment of conditions associated with impaired blood circulation, *e.g*. debilitated brain function, hearing loss, vertigo and tinnitus is a more recent development that started in western Europe [3]. The plant has a very good reputation for being effective against CNS problems [4–8].

The natural constituents of crude extracts of *Ginkgo biloba* include a group of alkyl phenols. They are mainly ginkgolic acids (GA), cardanols (*e.g*. ginkgol), and cardols (*e.g*. bilobol), which are largely eliminated from the commercial preparation of *Ginkgo biloba* for safety reasons, as they have been reported to exhibit an allergenic and possibly a genotoxic potential. For this reason, different monographs, such as the one issued by the Commission E of the former German Federal Health Agency or the European Pharmacopoeia, state that the maximal concentration should not exceed 5 ppm [9]. The alkyl phenols are eliminated during the multistep process of the standardized EGb 761^®^ preparation. They are separated and removed from the primary acetone extract as insoluble compounds (decanter sludge) [10, 11]. These compounds are known to inhibit the enzymatic activity of lipoxygenase, cyclooxygenase, aldose reductase, glucosidase and tyrosinase. In addition, they reveal antimicrobial effects and may, therefore, protect the plant from damaging environmental effects [10].

Some studies showed that the GA have potential anticancer activity. The viability of pancreatic cancer cells was suppressed by GA. In addition, GA induced apoptosis and impaired migration, invasion and the colony-forming capability of cancer cells [12–14]. GA also inhibited *de novo* lipogenesis in cancer cells by activation of AMP-activated protein kinase (AMPK) signaling and downregulation of several key enzymes involved in this process [14]. Additionally, GA inhibited the growth of HepG2 and Tca8113 cancer cells without affecting the growth of the non-cancerous cell line, MC-3T3-E1. Furthermore, GA induced apoptosis by down-regulation of BCL-2 and up-regulation of Bax [13]. They also suppressed lung cancer migration and invasion by inhibition of the PI3K/Akt/mTOR signaling pathway [12].

Sumoylation is a posttranslational modification process, in which a small peptide termed SUMO is covalently attached to the target protein at specific lysine residues. The SUMO-activating enzyme E1 (a heterodimer of SAE1 and SAE2) forms a strong thioester bond between the cysteine residue in the active site and the C terminal glycine of SUMO. SUMO is then directly transferred to the catalytic cysteine residue of the SUMO-conjugating enzyme E2 (Ucb9). Eventually, E2 recognizes the target protein and transfers SUMO to the epsilon amino group of lysine within a consensus site. Sumoylation can also be promoted by a group of enzymes termed SUMO ligase (E3) [15].

The process of sumoylation plays a vital role in the regulation of the NF-κB signaling pathways. The NF-κB essential modulator (NEMO) is a known substrate for SUMO modification. It plays a regulatory role in NF-κB signaling, through activating the degradation of nuclear factor of κ-light polypeptide gene enhancer in B-cells inhibitor α (IκBα) [16]. It is therefore expected that the inhibition of SUMOylation of NEMO suppresses the activation of NF-κB signaling in cells. GA and their analogue anacardic acid act as small molecule inhibitors of protein SUMOylation. They possibly inhibit sumoylation by the E1-SUMO thioester complex through direct binding to E1. Both the carboxylic acid and the aliphatic chain are essential for binding to E1 and the inhibitory effect on sumoylation [17].

Breast cancer represents a clinically heterogeneous disease. It is the leading cause of death in women and the second most common cancer worldwide [18]. The treatment options for breast cancer are quite versatile and often integrative, and these include systemic therapy (chemotherapy, hormonal therapy and biologicals), radiotherapy and surgical intervention [19]. Although breast cancer is a local disease, it can metastasize to local lymph nodes and later to distal organs. It is the metastasis at distal organs and tissues, but not the primary tumor that is main cause of death of patients. Around 10-15% of breast cancer patients appear with aggressive disease and develop metastasis within the first three years [20]. NF-κB is an important player in epithelial-mesenchymal transition (EMT) and the metastatic process. Inhibition of NF-κB signaling prevented EMT in breast cancer cells [21].

In this study, we investigated the anti-migratory effects of seven GA against two breast cancer cell lines and their effect on the expression of metastasis-related genes. We have also shown that the anti-migratory effects of GA were due to the suppression of NF-κB signaling and that the inhibition of NF-κB by GA can be explained by the inhibitory of sumoylation of regulatory proteins downstream of NF-κB.

## RESULTS

### GA inhibited wound healing in breast cancer cell lines without causing cytotoxicity

**Figure 1:**
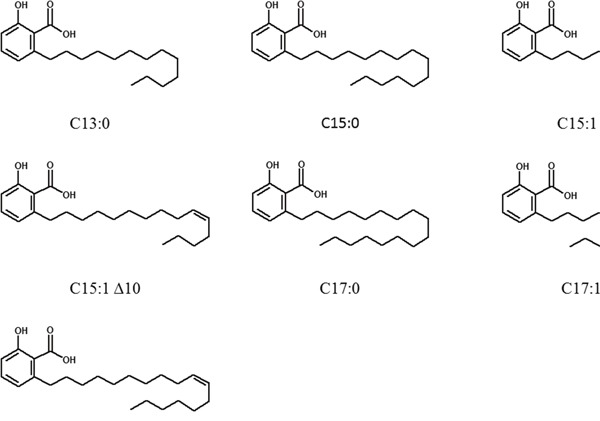
Chemical structures of ginkgolic acids

**Figure 2:**
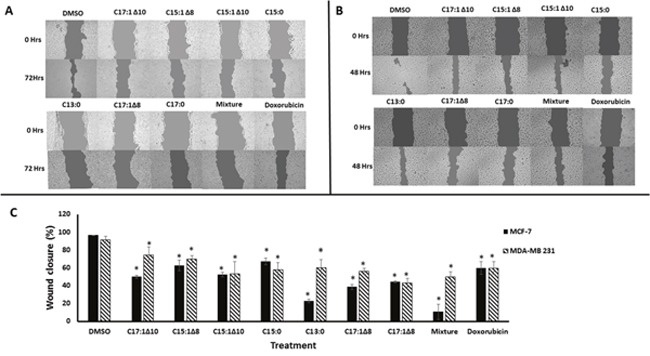
Ginkgolic acids inhibited breast cancer cell migration **(A)** Wound-healing assays taken at 0 and 72 h using MCF-7 cells treated with 25 μM of each GA. **(B)** Wound-healing assays taken at 0 and 48 h using MDA-MB 231cells treated with 25 μM of each GA. **(C)** Statistical quantification of the wound healing assays. DMSO was used as a negative control. Doxorubicin was used as a positive control drug. The results shown are the mean ± SD of at least three independent experiments. (*p < 0.05, compared to DMSO-treated control cells).

**Figure 3:**
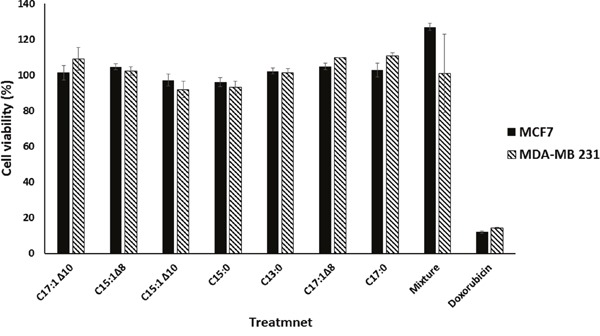
Ginkgolic acids did not affect the viability of breast cancer cells The bar chart illustrates the viabilities of breast cancer cells in the presence of 25 μM GA measured by the resazurin assay. Doxorubicin was used as a positive control drug. The results shown are the means ± SD of three independent experiments.

Wound-healing assays were performed using MCF-7 and MDA-MB 231 breast cancer cells to test the anti-migratory effects of the seven GA (Figure 1) and a mixture of them. Monolayer cells were grown to 100% confluence and then treated with GA. The wound-healing abilities of GA-treated cells were markedly inhibited (Figure 2). The GA mixture showed the best activity. These results suggest that GA significantly suppressed the migration of breast cancer cells. To exclude the possibility that the anti-migratory effects of GA were non-specific due to cytotoxic effects of the seven GA and a mixture of them, resazurin assays were performed at the same GA concentrations as that used for the wound-healing assays. As shown in Figure 3, neither the GA at 25 μM nor their mixture at 10 μg/ml showed any reduction in cell viability after 72 h treatment. These results suggest that the anti-migratory effects of the GA were not due to cytotoxicity caused by these compounds.

### GA downregulated NF-κB activity in a reporter cell line

**Figure 4:**
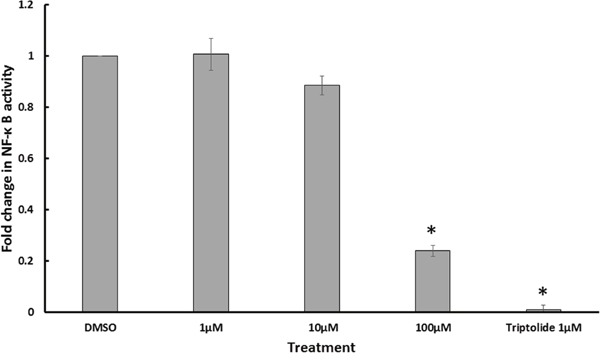
Ginkgolic acid C13:0 inhibited NF-κB activity NF-κB activity was determined by quantification of SEAP using Quanti Blue in TNF-α stimulated Heck Null Blue cells after 24 h of treatment. Triptolide, a known inhibitor of NF-κB activity, was used as positive control. Results shown are the mean values ± SD of three independent. (*p< 0.01, compared to DMSO control cells).

NF-κB pathway plays an important role in cancer progression and metastasis, especially in breast cancer [22]. In order to test the effect of GA on NF-κB activity, the reporter cell line HEK Blue Null 1 was treated with different concentrations of C13:0. Triptolide, which is known to inhibit the transcriptional activation of NF-κB [23], was used as a positive control. DMSO was used as a negative control. The NF-κB reporter activity was assessed by measurement of SEAP levels using Quanti-Blue. As shown in Figure 4, NF-κB activity was reduced in a dose-dependent manner with a significant reduction after treatment with 100 μM C13:0. The results suggest GA to be inhibitors of NF-κB activity.

### GA inhibited sumoylation of NEMO

**Figure 5:**
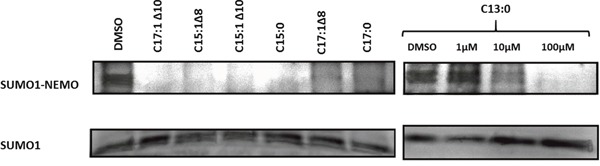
Ginkgolic acids inhibited SUMOylation of NEMO *in vitro* 100μM of C17:1 Δ10, C15:1Δ8, C15:1Δ10, C15:0, C17:1Δ8, C17:0 and 1μM, 10μM and 100μM of C13:0 were used. DMSO was used as a positive control. The sumoylation assay was performed with modified recombinant NEMO and a SUMOylation reaction mixture. Proteins were separated by SDS-PAGE and detected by Western blotting.

A key regulator of NF-κB activity is NEMO, a known target of SUMO [16]. Therefore, the effect of GA on the sumoylation of NEMO was investigated by the induction of sumoylation after treatment of recombinant NEMO with GA or DMSO. Sumoylation was induced by incubating NEMO in the presence of SUMO-1, SUMO E1, SUMO E2, Mg-ATP at 37°C for 1 h. The proteins were separated using SDS-PAGE and the sumoylation products were detected using the immunoblotting technique. As shown in Figure 5, it is obvious that all 7 GA inhibited sumoylation of NEMO. For C13:0, the effect appeared in a dose-dependent manner.

### GA reduced the degradation of IκBα

**Figure 6:**
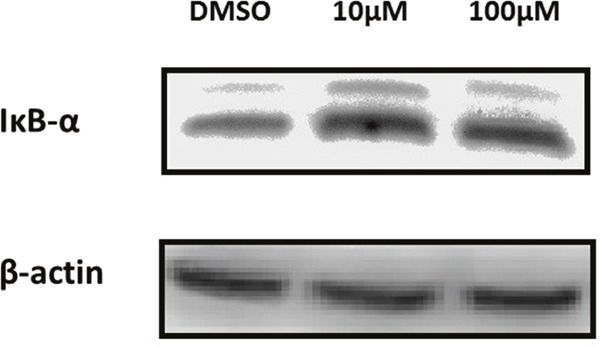
Ginkgolic acid C13:0 upregulated IκBα at the protein level in MDA-MB-231 cells Cells were treated with 10μM, 100μM or DMSO (control). Total protein was extracted and analyzed by Western blotting.

Activated NEMO, being part of the IKK complex, is responsible for the degradation of IκBα and consequently the activation of NF-κB [16]. In the aim of demonstrating the effect of GA on IκBα, we performed protein extraction, SDS-PAGE and Western blot analysis. Protein levels of IκBα have increased after treatment of MDA-MB-231 cells with C13:0 as shown in Figure 6, indicating inhibition of IκBα degradation.

### Molecular docking of GA on SUMO E1

**Figure 7:**
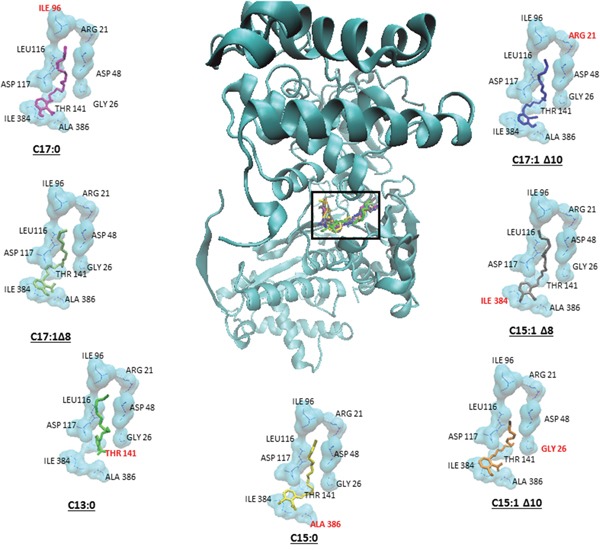
Binding of the seven ginkgolic acids at the adenylation domain of SUMO-activating enzyme E1 Each compound is shown in a different color. Hydrogen bond forming amino acids are shown in red.

GA is known to inhibit sumoylation by binding to and inhibiting SUMO activating enzyme E1 [17]. In order to determine the binding sites and the binding affinities, molecular docking was performed for GA on SUMO E1 by Autodock4 using the Lamarckian Algorithm. The results of the molecular dockings are shown in Figure 7. Table 1 shows the lowest binding energies, the numbers of interacting amino acids and the amino acids involved in hydrogen bonding. All seven GA showed high binding affinities with very low binding energies ranging from -10.28 to -12.27 kcal/mol. Additionally, all compounds bound to the same pharmacophore, *i.e*. the adenylation domain of the enzyme.

**Table 1:** Molecular docking results of ginkgolic acids on SUMO activating enzyme E1

	Ligand	Lowest binding Energy(kcal/mol))	No. of interacting amino acids	Amino acids showing H-bonds
**1**.	C17:1 Δ10	12.27 ± 0.19	9	ARG 21
**2**.	C15:1Δ8	11.04 ± 0.54	12	ILE 384
**3**.	C15:1 Δ10	11.44 ± 0.43	13	GLY 26
**4**.	C15:0	11.12 ± 0.37	13	ALA 386
**5**.	C13:0	10.28 ± 0.24	11	THR 141
**6**.	C17:1Δ8	11.46 ± 0.34	9	-
**7**.	C17:0	11.48 ± 0.79	8	ILE 96

### GA downregulate target genes of NF-κ B

**Figure 8:**
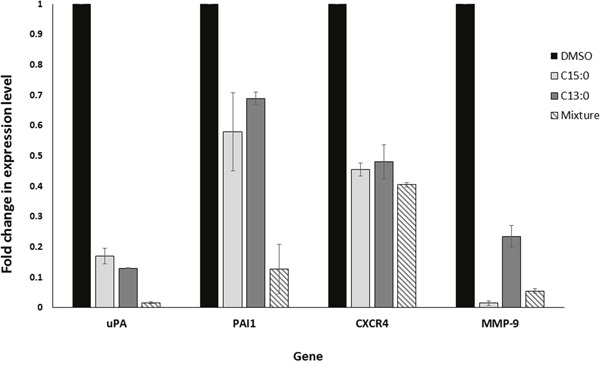
Ginkgolic acids downregulate the expression of the uPA, PAI-1, CXCR4 and MMP-9 genes Real time-PCR analysis was conducted for the four genes in untreated and GA-treated cells. The results were normalized by GAPDH. Fold changes were calculated with the 2^-ΔΔCt^ method.

Due to the inhibitory effect of GA on NF-κB, it is expected that they would in turn downregulate the expression of NF-κB target genes. RNA was extracted from MDA-MB-231 cells after treatment with C15:0, C13:0, the mixture, or DMSO. The levels of mRNA expression of NF-κB target genes uPA, PAI-1, CXCR4 and MMP-9, all of which strongly correlate with breast cancer metastasis [22, 24], were measured using quantitative real time PCR. As shown in Figure 8, all four genes were downregulated after treatment with the GA. The mixture was found to be more effective than isolated C15:0 and C13:0.

## DISCUSSION

Breast cancer is the leading cause of death among women in developed countries [18]. It includes multiple subtypes with distinct morphologies and clinical manifestations [25]. About 90% of cancer-related deaths are related to metastasis [26]. Metastasis is the process, where malignant cells migrate from the primary tumor and establish a secondary metastatic colony in another organ. This process requires the migration of tumor cells into the surrounding tissues and their eventual entry into the blood stream to reach the secondary site [27]. Therapeutic goals in cancer treatment include not only the eradication of tumors from their primary site, but also the prevention of an initial metastasis in high-risk patients, curbing already established lesions and the prevention of additional metastases [28]. Therefore, the suppression of metastasis may result in the improved survival times and better prognosis for breast cancer patients.

Our objective in this study was to analyze the possible effects of GA on the invasiveness and migratory ability of breast cancer cells and to identify the possible mechanisms for their action. Previous studies have shown that GA exhibit promising antitumor activities. The wound-healing and anti-migratory effects on pancreatic and lung cancer have been previously described [12, 14]. We found that GA have anti-migratory effects on the triple-negative MDA-MB-231 breast cancer cell line as well as in the less invasive MCF-7 cells. These effects were not related to the cytotoxic effects of GA, as the wound healing and possible anti migratory effects appeared at concentrations that were non-cytotoxic to both cells. Various mechanisms have been reported, regarding the cytotoxic and anticancer activities of GA. They activate protein phosphatase 2C (PP2C) [4] and inhibit fatty acid synthase (FAS) [29], and α-glucosidase [30]. In addition, GA inhibited *de novo* lipogenesis in cancer cells by activating AMPK signaling and lowering the expression of enzymes involved in lipogenesis, including acetyl-CoA carboxylase (ACC), fatty acid synthase (FASN) [14]. Therefore, it appears that GA target several pathways in cancer cells.

We found that GA inhibited NF-κB reporter activity. The NF-κB pathway plays an important role in cancer progression and metastasis, especially in breast cancer. NF-κB up-regulates the expression of matrix metalloproteinases (MMP), urokinase-type plasminogen activator (uPA), and cytokine receptors in highly metastatic breast cancer cell lines [22]. Furthermore, NF-κB regulates the motility of breast cancer cells by directly up-regulating the expression of CXCR4. Overexpression of IκB in breast cancer cells with constitutive NF-κB activity resulted in reduced expression of CXCR4 and a corresponding loss of SDF-α -mediated migration *in vitro*. We, therefore, postulate that GA are inhibitors of NF-κB activity, and that this inhibition is at least in part responsible for their anti-migratory activity.

Furthermore, we found that GA reduce the degradation of inhibitory IκB protein, which interacts with and tightly regulates NF-κB activity. NF-κB is normally present as an inactive, IκB-bound complex in the cytoplasm. In both the canonical and non-canonical NF-κB activation pathways, the common upstream regulatory step represents the activation of the IκB kinase (IKK) complex, which consists of (IKKα and/or IKKβ) subunits and NF-κB essential modulator (NEMO). This complex phosphorylates and degrades the inhibitor IκB leading to the activation of NF-κB dimers. The NF-κB dimers eventually enter the nucleus and activate specific target gene expression [31]. Hence, NEMO can be considered as potential target for the downregulation of NF-κB activity, as it is absolutely required for both the canonical and non-canonical activation pathways.

**Figure 9:**
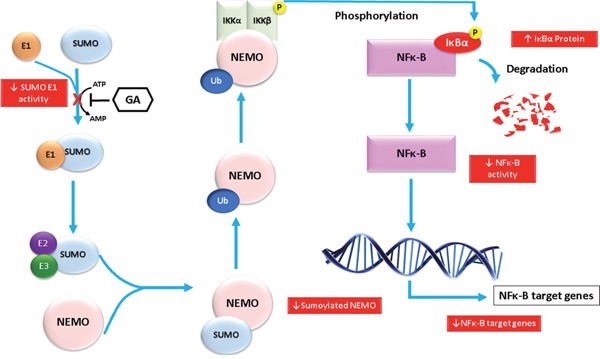
Diagram showing the molecular effects of ginkgolic acids on the NF-κB pathway that appeared in the study The molecular effects are shown in red boxes.

Sumoylation assay for NEMO with different concentrations of GA was used to justify our findings on the NF-κB inhibitory activity. Evidence from previous studies have shown that the modification of NEMO by SUMO prior to its accumulation in the nucleus is required for NF-κB activation [16]. In addition, the PIDD and RIP1 proteins were associated with, and thereby favor, the sumoylation of NEMO [32, 33]. After conducting sumoylation assays for NEMO using different concentrations of GA, we found a reduction of sumoylated NEMO levels at concentration of 10 and 100 μM. A previous study showed that GA inhibited sumoylation of RanGAP1-C2 and p53 both *in vitro* and *in vivo*. These findings support our results regarding sumoylation of NEMO. Consequently, the inhibitory effects of GA on NF-κB activity can be explained by the inhibition of sumoylation of NEMO leading to the failure of the IKK complex formation and IκB degradation. The suggested mechanism is illustrated in Figure 9. Earlier studies demonstrated that NEMO undergoes transient modification with SUMO-1 dependent on its zinc finger domain. Inspection of the NEMO sequence identified two lysine residues (Lys 277 and Lys 309) located within a consensus SUMO modification motif [16]. To allow NF-κB activation, NEMO has to be then phosphorylated by ATM on Lys 85, leading to its monoubiquitination and to the export of a NEMO–ATM complex out of the nucleus [34]. Relocalization of ubiquitinated NEMO to the cytoplasm then allows activation of the IKK complex. We have used the polyubiquitin chain binding domain of NEMO (amino acids 183-339). Therefore, the two lysine residues involved in sumoylation of NEMO are therefore included in the polypeptide domain used.

We used molecular docking to predict the binding affinities and the binding sites of GA on SUMO activating enzyme E1 (SUMO E1). In the post translational modification process of sumoylation, SUMO is covalently attached to the target protein on specific lysine residues [35]. The SUMO activating enzymes E1 and E2 are required for this process. Evidence from binding assays with a fluorescently labeled probe showed that GA directly binds SUMO-activating enzyme E1 and inhibits the formation of the E1-SUMO intermediate [17]. However, the exact binding site and the binding affinities of GA on SUMO have not yet been investigated. Our results from molecular docking have shown that GA showed very low binding energies (between -10.28 and -12.27 kcal/mol), which implies that all ligands would show high binding affinities to SUMO E1, if tested *in vitro*. More interestingly all GA docked on the same pharmacophore and interacted with the same amino acids. Six of the seven GA showed interaction with hydrogen bonds. The pharmacophore, where the GA docked, lies in the Sae2 adenylation domain (amino acids 1–158, 384–438) [35].

Using quantitative RT-PCR, we investigated the effects of GA on the mRNA expression levels of the metastasis-related genes uPA, PAI-1, CXCR4 and MMP-9. We found that GA downregulate the expression of all four metastasis related genes. These genes are known to promote tumor progression and metastasis [22, 36, 37], and are associated with poor prognosis in breast cancer patients [22, 38–40]. uPA is an extracellular matrix-degrading protease involved in cancer invasion and metastasis that interacts with PAI. Breast cancer patients with high levels of u-PA and PAI-1 had significantly higher relapse rates than those with low levels [41]. The SDF-1/CXCR4 signaling pathway is involved in the growth and proliferation of breast cancer cells. Tumors from patients with high levels of CXCR4 metastasized more extensively than to those with lower levels [42]. Matrix metalloproteinases, a family of zinc-dependent endopeptidases are responsible for remodeling the extracellular matrix [43]. MMP-9 was found to be up-regulated in breast cancer samples. Furthermore, its expression was positively associated with lymph node metastasis and lymphangiogenesis in breast cancer [40]. uPA, PAI-1, CXCR4 and MMP-9 are known downstream targets of the NF-κB transcription factor [22, 24, 44–48]. It is therefore quite reasonable to assume that the down-regulation of these genes is the result of the inhibition of NF-κB activity.

In conclusion, GA showed significant anti-migratory effects, and therefore, have a great potential to control the metastatic dissemination of breast cancer cells. For the first time, we have shown that GA significantly inhibited the NF-κB activity, most probably by blocking the sumoylation of NEMO. The NF-κB target genes uPA, PAI-1, CXCR4 and MMP-9 were found to be downregulated. These results suggested that GA might reveal anti-metastatic and anti-EMT effects. Further experiments are required to confirm this result. Although GA are known to possess a contact allergic potential, they can be considered as interesting non-cytotoxic anticancer agents, from which breast cancer patients might benefit, especially if used in combination with other anticancer drugs.

## MATERIALS AND METHODS

### Cell-lines and reagents

MCF-7 cells were obtained from the German Cancer Research Center (DKFZ, Heidelberg, Germany). The original source of the cell lines is the American Type Culture Collection (ATCC, USA) German Cancer Research Center, Heidelberg, Germany). MDA-MB-231 cells were kindly provided by Dr. W. K. Cavenee (Ludwig Institute for Cancer Research, San Diego, CA). Both cell lines were grown in DMEM supplemented with 10% fetal bovine serum (FBS), penicillin (100 U/ml)/streptomycin (100 μg/ml). HEK Blue Null 1 cells were maintained at 37°C and 5% CO_2_ in DMEM supplemented with 4.5 g/l glucose, 4 mM L-glutamine, 10% fetal bovine serum (FBS), 100 U/ml penicillin, 100 U/ml streptomycin 100 μg/ml Normocin (Invivogen, Toulouse, France) and 100 U/ml Zeocin (Invivogen).

### Antibodies

A primary antibody against IκBα and a secondary anti-mouse antibody were purchased from Cell Signaling, Technology/New England Biolabs (Frankfurt, Germany). Primary antibody against SUMO-1 was purchased from Enzo Life Sciences GmbH (Lörrach, Germany).

### Compounds

A panel of seven GA (C13:0, C15:0, C15:1Δ8, C15:1Δ10, C17:0, C17:1Δ8 and C17:1Δ10) and a mixture of total GA from *Ginkgo biloba* raw extract were kindly provided by Dr. Willmar Schwabe GmbH & Co. KG (Karlsruhe, Germany). The chemical structures of the seven GA are shown in Figure 1. Triptolide was purchased from Invivogen.

### Wound-healing assay

Using MCF-7 and MDA-MB-231 cells, 1× 10^6^ cells/well were seeded in 6-well plates and allowed to grow overnight to confluent monolayers. The cell monolayers were carefully scraped with a sterile 100 μl pipet tip to create a scratch. Subsequently, cells were washed with PBS and treated with DMEM culture medium containing 25 μM of each of the seven GA, 0.01 mg/ml of the mixture, 0.6 μM doxorubicin (positive control drug) or 0.5% DMSO (solvent control). Images of the scratches were taken after 0 and 72 h for MCF-7 cells and after 0 and 48 h for MDA-MB 231 using a Juli-Br cell analyzer (NanoEnTek Inc., Seoul, Korea). Data analysis was performed with TScratch software (CSEI lab-ETH, Zurich, Switzerland).

### Screening for cytotoxicity

MCF-7 and MDA-MB 231 cells obtained from exponential phase cultures by were counted and seeded into 96-well plates. The seeding density was 10^4^ cells/well for both cell lines. Cells were then treated with 25 μM of each of the seven GA, 0.01 mg/ml of the mixture, 0.6 μM doxorubicin (positive control) or 0.5% DMSO (solvent control). After incubation for 72 h, 20 μl of resazurin 0.01% w/v were added to each well and the plates were incubated at 37°C for further 4 h. The fluorescence was measured on an Infinite M2000 Pro plate reader (Tecan, Crailsheim, Germany). Compounds were defined as active, if the mean cell viability was less than 50%. The viability was evaluated based on a comparison with untreated cells.

### NF-κB reporter assay

HEK Blue Null 1 cells (Invivogen) were treated with three different concentrations of GA C13:0 for 24 h and then activated with TNF-α for another 24 h. NF-κB activation was detected after the addition of Quanti Blue (InvivoGen) by measuring the levels of secreted alkaline phosphatase (SEAP) at 630 nm using an Infinite M2000 Pro plate reader. Triptolide was used as a positive control compound. Three independent experiments were performed.

### Protein extraction

MDA-MB 231 cells were grown in 175 cm^3^ flasks and treated with C13:0 at 10 μM, 100 μM or DMSO control for 24 h. Cells were then washed once with PBS. M-PER® Mammalian Protein Extraction Reagent, including protease inhibitor, was added to each flask. Cells were harvested using a cell scraper and transferred to a 2 ml Eppendorf tube. The cell solution was shaken for 30 min at 4 °C. Samples were centrifuged (14000 × g, 15 min, 4 °C) and supernatants were transferred to new Eppendorf tubes and the protein concentrations were determined using NanoDrop1000 (Thermo Fisher Scientific, Wilmington, DE, USA). Thirty micrograms of protein were taken for each sample and analyzed by SDS-PAGE and Western blotting.

### SUMOylation assay

An *in vitro* sumoylation kit (Enzo Life Sciences, Lörrach, Germany) was used to investigate the effect of C13:0 on the sumoylation of NEMO. 1, 10 and 100 μM of the compound or DMSO (solvent control) were added to the *in vitro* sumoylation reaction mixture prepared according to the manufacturer's recommendations. The mixture included SUMO-1, SUMO E1, SUMO E2, Mg-ATP complex and 200 nM NEMO (Ubiquitin-Proteasome Biotechnologies, Aurora, USA). A mixture without Mg-ATP complex was used as negative control. The mixtures were incubated at 37°C for 60 min. Samples were then analyzed by SDS-PAGE and Western blotting.

### SDS-PAGE and western blot analysis

SDS-loading buffer was added to protein from each sample. The mixture was incubated at 95°C for 10 min and loaded onto an SDS-polyacrylamide gel. Proteins were then transferred by Western blotting on a PVDF membrane. The membrane was blocked with BSA/TBS-T solution and probed with primary antibody (1:1000) followed by HRP-linked secondary anti-rabbit or anti-mouse IgG antibody (1:2000). Proteins were detected using Luminata Classico HRP Western Blot substrate (Merck Millipore, Schwalbach, Germany). The membrane was visualized and documented with an Alpha Innotech FluorChem Q system (Biozym, Oldendorf, Germany).

### Molecular docking

The 2D structures of the seven GA were drawn using ChemSketch (Advanced Chemistry Development, Inc., Toronto, Canada) and converted into 3D structures using Open Babel 2.3.1. The PDB file for SUMO E1 activating enzyme (PDB ID:1Y8Q) was downloaded from the Protein Data Bank (http://www.rcsb.org/pdb/). To perform molecular docking, the protein structure of SUMO E1 was first processed with AutodockTools-1.5.6rc316 to overcome problems of incomplete structures due to missing atoms or water and the presence of multimers or interaction partners of the receptor molecule. The output file after preparation was in PDBQT format, where information about atomic partial charges, torsion degrees of freedom and different atom types were added, *e.g*. aliphatic and aromatic carbon atoms or polar atoms forming hydrogen bonds. A grid box was then constructed to define docking spaces. The dimensions of the grid box were set around the whole SUMO E1 molecule as such that the ligand could freely move and rotate in the docking space. The grid box consisted of 126 grid points in all three dimensions (X, Y and Z) separated by a distance of 1 Å between each one. Energies at each grid point were then evaluated for each atom type present in the ligand, and the values were then used to predict the energy of a particular ligand configuration. Docking parameters were set to 250 runs and 2,500,000 energy evaluations for each cycle. Docking was performed for each ligand on SUMO E1 by Autodock4 using the Lamarckian Algorithm. The corresponding binding energies and the number of conformations in each cluster were attained from the docking log files (dlg).

### Primers

Primers for uPA, PAI-1, CXCR4 and MMP-9 were designed using Primer3 (http://bioinfo.ut.ee/primer3-0.4.0/primer3/). The MFEprimer-2.0 online tool (http://biocompute.bmi.ac.cn/CZlab/MFEprimer-2.0/) was used to test primer specificity and usability. Amplification specificities were rechecked with Primer Blast (http://www.ncbi.nlm.nih.gov/tools/primer-blast) using the sequence data from the NCBI RefSeq Human mRNA data base (http://www.ncbi.nlm.nih.gov/refseq/). The housekeeping gene GAPDH was used as a reference for normalization. Primers were synthesized by Eurofins MWG Operon. The sequences of the primers are shown in Table 2.

**Table 2:** Foreword and reverse primers used for quantitative RT-PCR

	Gene	Forward primer	Reverse primer
**1**.	uPA	GCCATCCCGGACTATACAGA	ACACAGCATTTTGGTGGTGA
**2**.	PAI-1	CTCTCTCTGCCCTCACCAAC	GTGGAGAGGCTCTTGGTCTG
**3**.	CXCR4	CCGTGGCAAACTGGTACTTT	TTCCTTGGCCTCTGACTGTT
**4**.	MMP-9	TTGACAGCGACAAGAAGTGG	GCCATTCACGTCGTCCTTAT
**5**.	GAPDH	TGGGCTACACTGAGCACCAG	GGGTGTCGCTGTTGAAGTCA

### RNA extraction and cDNA synthesis

MCF-7 and MDA-MB 231 cells were treated for 24 h with 25 μM C15:0, 25 μM C13:0, 0.01 mg/ml of the mixture or DMSO solvent control. Total RNA was isolated using InviTrap® Spin Universal RNA Mini kit (Stratec Biomedical, Birkenfeld, Germany) according to the manufacturer's instructions. The RNA extracted was converted to cDNA with random hexamer primers using RevertAid H Minus First Strand cDNA Synthesis Kit (Thermo Scientific, Darmstadt, Germany).

### Quantitiative RT-PCR

Quantitiative RT-PCR was performed using CFX384™ Real-Time PCR Detection System (Bio-Rad, Munich, Germany). Briefly, 4 μl 5× Hot Start Taq EvaGreen qPCR Mix (Axon, Kaiserslautern, Germany), 250 nM final primer concentration and 300 ng RNA (converted to cDNA) were used per reaction in a total volume of 20 μl. Real-time RT-PCR was performed as follows: initial denaturation at 95 °C for 10 min, 40 cycles including strand separation at 95 °C for 10 sec, annealing at 55 °C for 15 sec and extension at 72 °C for 40 sec following final extension at 95 °C for 1 min. All measurements were done in duplicates and the experiment was repeated twice. Standardized Ct (cycle threshold) values for the genes in samples were obtained by dividing the Ct values of genes in GA treated samples by Ct values of GAPDH gene in treated samples and multiplying with the Ct value of GAPDH in the DMSO control. Fold changes were calculated with the ΔCt (Standardized Ct of the gene in drug-treated sample - Ct of the gene in DMSO control) method where the fold change is equal to 2^-ΔΔCt^.

### Authors' contributions

Sami Hamdoun performed the experiments and wrote the paper. Thomas Efferth supervised the project and wrote the paper.

## Abbreviations

EMT: epithelial-mesenchymal transition
GA: ginkgolic acids
SUMO: small ubiquitin-related modifier
NF-kB: nuclear factor kappa-light-chain-enhancer of activated B cells
NEMO: NF-kappa-B essential modulator
IκBα: nuclear factor of kappa light polypeptide gene enhancer in B-cells inhibitor alpha
uPA: urokinase plasminogen activator
PAI-1: plasminogen activator inhibitor-1
CXCR4: C-X-C chemokine receptor type 4
MMP-9: matrix metalloproteinase 9
